# Dr. Stuart Harry Benton

**Published:** 1915-04

**Authors:** 


**Dr. Stuart Harry Benton,** Buffalo, 1870; Bellevue Hospital Medical College, 1879; died at his home in Brooklyn, March 2, aged 69.

